# Li-Ca Alloy Composite Anode with Ant-Nest-Like Lithiophilic Channels in Carbon Cloth Enabling High-Performance Li Metal Batteries

**DOI:** 10.34133/2022/9843093

**Published:** 2022-06-23

**Authors:** Zihao Wang, Yuchi Liu, Jianxiong Xing, Zhicui Song, Aijun Zhou, Wei Zou, Fu Zhou, Jingze Li

**Affiliations:** ^1^ School of Materials and Energy, University of Electronic Science and Technology of China, Chengdu 611731, China; ^2^ Yangtze Delta Region Institute (Huzhou), University of Electronic Science and Technology of China, Huzhou 313001, China; ^3^ Research and Development Center, Tianqi Lithium Co., Ltd., Chengdu 610093 China

## Abstract

Constructing a three-dimensional (3D) multifunctional hosting architecture and subsequent thermal infusion of molten Li to produce advanced Li composite is an effective strategy for stable Li metal anode. However, the pure liquid Li is difficult to spread across the surface of various substrates due to its large surface tension and poor wettability, hindering the production and application of Li composite anode. Herein, heteroatomic Ca is doped into molten Li to generate Li-Ca alloy, which greatly regulates the surface tension of the molten alloy and improves the wettability against carbon cloth (CC). Moreover, a secondary network composed of CaLi _2_ intermetallic compound with interconnected ant-nest-like lithiophilic channels is in situ formed and across the primary scaffold of CC matrix by infiltrating molten Li-Ca alloy into CC and then cooling treatment (LCAC), which has a larger and lithiophilic surface to enable uniform Li deposition into interior space of the hybrid scaffold without Li dendrites. Therefore, LCAC exhibits a long-term lifespan for 1100 h under a current density of 5 mA cm ^-2^ with fixed areal capacity of 5 mAh cm ^-2^. Remarkably, full cells paired with practical-level LiFePO _4_ cathode of 2.45 mAh cm ^-2^ deliver superior performance.

## 1. Introduction

Lithium (Li) metal is regarded as the most proposing material of anode for the next-generation high-energy-density batteries due to its ultrahigh theoretical specific capacity (3860 mA h g ^-1^) and the lowest reduction potential (-3.04 V versus the standard hydrogen electrode). However, Li dendrites growth and the “infinite” volume change during cycling hinder its application [ 1– 3]. Generally, constructing an architectural host and then infiltrating molten Li metal into a three-dimensional (3D) conductive skeleton to alleviate the volume change is regarded as an effective strategy to enhance the lifespan and stability of Li metal anode [ 4– 8]. Among various 3D conducting skeletons, carbon-based materials are very promising as advanced host of Li metal anode due to the benefits of low density, favorable electrical conductivity, cheap, excellent chemical stability, and robust mechanical strength [ 9– 13].

Recently, researchers have explored the application of carbon-based skeletons in Li metal anodes [ 14– 19]. The construction of self-supported carbon fibers network is an ideal choice for 3D carbon-based host. For example, Zuo et al. [ 19] self-assembled graphitized carbon fibers (GCF) as a collector for Li metal anode. The obtained GCF not only alleviates the volume change during Li plating/stripping process but also accommodates the 8 mA h cm ^-2^ Li deposition, suggesting that carbon-based material is one of the best choices for hosting Li anode with high areal capacity.

Alternatively, it is significant to develop a smart and facile method for prestoring Li inside porous 3D carbon-based scaffolds to create Li composite. Many thermal infusion strategies have been developed [ 20– 22]. Nevertheless, liquid Li has high surface tension due to its strong internal metal bonds, which prevents the spreading of the molten Li onto carbon-based scaffold or leads to so-called poor wettability [ 23– 25]. Therefore, reducing the surface tension of liquid Li to enhance its wettability is critical to fabricate advanced Li composite. In addition, the hazards of large Li nucleation overpotential on carbon-based matrix also need to be solved [ 26, 27]. There are two critical strategies to lower surface tension and Li nucleation overpotential. The first one is to introduce reactive species to promote Li wettability of carbon-based materials [ 28– 36]. Niu et al. [ 28] found that carbon hosts can transfer from nonwetting to superwetting toward Li after an amine functionalized treatment. Consequently, Li metal can grow along the carbon fibers during cycling, and preferential nucleation occurs within the mesopores in the carbon fiber, inhibiting Li dendrite generation effectively. Similarly, Huang et al. [ 30] fabricated a nitrogen- (N-) doped 3D porous graphene as the host for Li metal anode, which consists of smoothly interconnected graphene sheets in a 3D porous nanostructure. Benefiting from the functional groups generated on the graphene surface after N-doping, molten Li can be easily infiltrated into the host structure within 1 min due to the lower surface tension. These findings exhibit the significance of introducing functional groups into the carbon-based substrates. However, the problem of the functional groups’ degradation on the carbon-based materials during cycling is still not resolved well [ 35, 37]. On the other hand, lithiophilic metal/nonmetal-based coating species which can react with Li to form alloys or metallic compounds on the carbon-based host and thus lower surface tension of liquid Li have been screened to tune the Li wettability [ 38– 50]. Li et al. [ 43] constructed Co _3_O _4_ nanofibers on carbon sheets (CS). The changes of free energy ΔG of the reaction between Li metal and Co _3_O _4_ are negative, resulting in the reaction of molten Li metal with Co _3_O _4_ and the formation of Li _2_O/Co, thereby reducing the surface energy of the matrix. And the Co _3_O _4_ nanofibers vertically grow on the carbon fiber substrate, providing confined space for Li deposition and effectively redistributing Li ^+^ flux to guide uniform Li nucleation. Furthermore, Zhang et al. [ 46] coated the carbon fibers with a SnO _2_ layer and then infiltrated molten Li to form a 3D composite anode within 27 s. Each carbon fiber with the coaxial structure includes an electronic conductive carbon core and an alloy buffer layer with excellent Li wettability as well as an adherent Li metal shell layer. Remarkably, the Li-Sn alloy is an electron/ion mixed conductor with a higher Li diffusion coefficient, endowing faster Li nucleation and SEI formation kinetics. Similarly, Wang et al. [ 50] constructed ZnO nanofiber on carbon cloth (CC) and then improved the wettability of CC for infiltration of molten Li. Notably, the Li _2_O insulating layer is formed in situ during the process of Li infiltration, which may screen the Li wettability due to the high formation energy of the Li _2_O/Li interfaces and increase the interfacial impedance [ 51, 52].

More significantly, it is well-known that the areal capacity of commercial Li-ion battery is about 3 mA h cm ^-2^, which is almost equal to 15 *μ*m thick Li metal deposition. Generally, the thickness of the common coating layer on the host does not exceed few micrometers. Therefore, the lithiophilic species with limited specific surface area can be rapidly buried by the plated Li layer, and then, the Li metal may grow uncontrollably on the surface of 3D host, which is a key reason for the limited performance of the Li composite anode [ 53, 54]. Therefore, the introduction of reactive species to enhance Li wettability has limitation for practical application as mentioned above. Thus, doping heterogeneous elements into molten Li to prepare Li-rich alloy has been challenged for decreasing surface tension [ 23, 55– 57]. For example, Wang et al. [ 55] found that liquid Li-Sn alloy has favorable wettability on various substrates and can be infiltrated into garnet pellets less than 10 s. Hence, there is no doubt that alloying approaches are highly facile and applicable to carbon-based substrates for lowering surface tension and guide uniform Li deposition. Furthermore, Li-rich dual-phase alloy is featured with a self-assemble microporous 3D scaffold with larger specific surface area, which can regulate Li deposition morphology even at a large areal capacity [ 7, 58]. One of the promising candidates is dual-phase Li-Ca alloy which has excellent lithiophilicity and a typical pattern of interconnected microporous framework named as ant-nest-like network.

Based on this idea, we prepared ant-nest-like Li-Ca alloy network showing good lithiophilicity hosted in CC matrix by a simple thermal infiltration treatment (named as LCAC). The introduction of heterogeneous Ca metal into molten Li significantly reduces the surface tension due to the lower bonding force between Ca and Li [ 23, 25], enhancing the wettability and accelerating the diffusion speed of molten Li into CC matrix. The molten Li-Ca alloy forms the interconnected microporous structure composed of CaLi _2_ intermetallic compound in situ during the cooling process, which acts as a secondary network distributed in the CC uniformly. Remarkably, the Li-Ca alloy microporous framework not only provides larger surface area and lower local current density for Li nucleation but also effectively assists uniform Li deposition on the alloy scaffold to inhibit the growth of Li dendrites effectively. Additionally, while the CaLi _2_ intermetallic compound is a MgZn _2_ type Laves phase structure with less flexible and lower fracture toughness, the good flexibility of CC can significantly solve this problem and improve the mechanical stability of the Li-Ca alloy [ 58]. As a result, the LCAC anode exhibits better electrochemical performance, demonstrating an ultralong stable cycling performance up to 3500 h in the configuration of a symmetric cell with a current density of 1 mA cm ^-2^ and areal capacity of 1 mA h cm ^-2^. Even at the higher current density and areal capacity to 5 mA cm ^-2^ and 5 mA h cm ^-2^, the cyclic lifetime is more than 1100 h. In a full cell assembled with large areal capacity lithium iron phosphate (LFP) cathode of 2.45 mAh cm ^-2^, the capacity can retain about 94% after 1000 cycles at a rate of 1 C.

## 2. Results and Discussion

**Figure 1:**
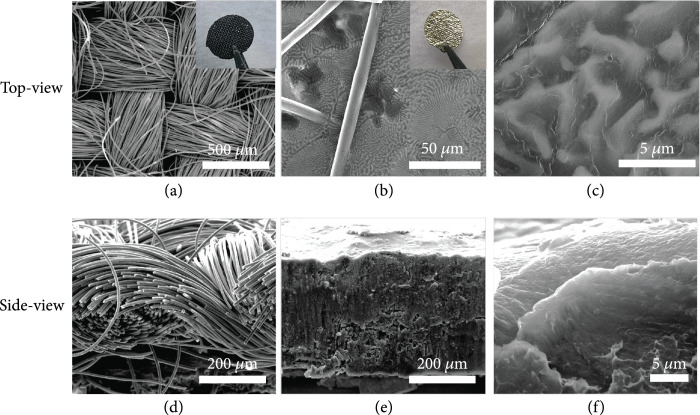
The top-view SEM images of the (a) CC and the (b, c) LCAC with the corresponding optical photographs (insert images). The side-view SEM images of the (d) CC and the (e, f) LCAC.

The CC was obtained by commercial channels and used as the Li host in terms of the following considerations. (1) The CC is cheap, which can facilitate future industrial mass production for practical application; (2) benefiting from excellent mechanical properties and high electrical conductivity, the CC as the primary host can greatly improve mechanical strength and electrochemical performance of Li-Ca alloy composite anode; (3) the CC is much lighter than the metallic framework, rendering relatively higher energy density. The weaved network of CC can be distinctly exposed via SEM imaging (Figure 1(a)). Each bundle of carbon fibers consisting of hundreds of single fibers is compact weaved together, ensuring the structural integrity of CC. The cross-sectional SEM image exhibits the interweaved arrangement of carbon fibers, providing sufficient space to accommodate Li deposition (Figure 1(d)). The optical photograph shows a black and lusterless morphology. Then, the pristine CC is immersed into the molten Li and molten Li-Ca alloy so as to explore their wettability at 400°C. As demonstrated in Fig. S1a, the liquid Li shows unfavorable poor wettability toward CC, which cannot infuse into CC hosting matrix within 200 s. This result evidences the large surface tension of liquid Li. Therefore, we need to elevate the temperature up to 500°C for fabricating LiC composite successfully. By deep contrast, when the CC sheet comes into connect with liquid Li-Ca alloy at 400°C, the silvery molten Li-Ca alloy can wet the CC and fill up the whole porous skeleton completely less than 60 s. This favorable wettability is attributed to the doped Ca atoms lowering the surface tension of the as-formed Li-Ca alloy, in which a negative Gibbs free energy change is the driving force for the alloying reaction between metallic Ca and Li, leading the weaker bonding force between Ca and Li atoms with respect to that between Li and Li atoms [ 23, 56]. In order to clearly demonstrate the effect of heteroatomic elements doping on the wettability of molten Li, Li-Ca and Li-Ag alloys with various molar ratios were tested on CC substrate. As shown in Fig. S2, both of molten Li-Ca and Li-Ag binary alloys show much improved wettability at different ratios compared to the molten Li. When the atomic ratio of Ca/Li and Ag/Li approaches 1/25, the molten alloys exhibit the strongest binding affinity toward CC substrate. After the molten Li-Ca alloy is rapidly infiltrated and subsequently cooled down, the surface of the carbon cloth is composed of Li-Ca alloy micropore network with a diameter of 2-3 *μ*m (Figures 1(b) and 1(c)). And the skeleton profile of the CC can still be identified with a golden metallic luster. As shown in the corresponding side-view SEM imaging (Figure 1(e)), the Li-Ca alloy fills the whole CC skeleton densely. The pore size of the CC matrix is greatly decreased, and the specific surface area of the skeleton is greatly enlarged, providing more Li nucleation sites and reducing the current density more effectively to inhibit Li dendrite formation according to Sand’s law.

In order to analyze the phase structure of LCAC, an ex situ XRD measurement was tested, and the pattern of results is shown in Fig. S3. The LCAC consists of metallic Li, CaLi _2_, and carbon three components, and their mass percentages are 47.96%, 16.19%, and 35.85%, respectively (Table S1). This suggests that the ant-nest-like network is mainly composed of CaLi _2_, which agrees with the previous observation on Li-Ca dual-phase alloy [ 58]. Obviously, the LCAC composite anode has a high specific capacity since the content of Li metal phase is dominated. As shown in Fig. S4, after Li is stripped at 2 V, LCAC offers a specific capacity of about 1954 mA h g ^-1^, which is nearly equal to the theoretical specific capacity of 2011 mA h g ^-1^. Furthermore, LiC composite electrode is fabricated by infiltrating molten Li into CC at 500°C. As shown in Fig. S5a, the skeleton profile of CC is completely covered by Li metal in terms of the optical photograph. The corresponding cross-sectional SEM (Figs. S5c-d) shows a loose morphology, indicating that the condensed Li is weakly coated on the CC surface. This phenomenon reflects the poor wettability between Li and CC. In addition, the structure stability and morphology of the subskeleton nested in CC skeleton were investigated after the LCAC was stripped of 20 mA h cm ^-2^ Li at the current density of 1 mA cm ^-2^ (Fig. S6). The CaLi _2_ micro/nanosized scaffolds are tightly attached to each single carbon fiber, which are randomly aligned and form interconnected channels for efficient electron and ion transport. Then, the fully delithiated LCAC was prepared while the stripping voltage was further tuned down to -1 V. The surface morphology is shown in Figure S7a-b. Apparently, the secondary scaffold is stably adhered to the carbon fibers. The macroscopic CC framework and microscopic secondary scaffold are successfully integrated as the multiscale and multifunctional 3D network, in which the subscaffold enlarges the specific surface area and provides numerous lithiophilic sites to guide Li plating conformally. The corresponding XRD pattern (Fig. S7c) manifests that the fully delithiated alloy is solely composed of metallic Ca, implying that the alloying/dealloying reaction between Li and Ca is highly reversible, which is consistent with our previous observation [ 58]. In a sentence, the CaLi _2_/Ca framework is bound together with the carbon fibers and can be further self-assembled into ant-nest-like network, enhancing structural stability and lithiophilicity of CC skeleton for the improved performance.

**Figure 2:**
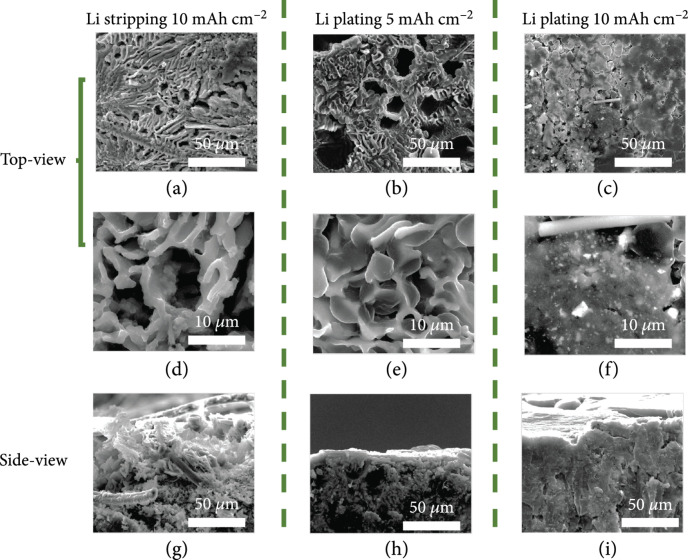
The SEM images of LCAC anode after (a, d, g) stripping 10 mA h cm ^-2^ Li, (b, e, h) plating 5 mA h cm ^-2^ Li back, and (c, f, i) plating 10 mA h cm ^-2^ Li back.

To investigate behavior of Li stripping/plating and the structure and morphology evolution of the LCAC composite anode, the metallic Li was first stripped and then replated at the current density of 1 mA cm ^-2^ in the LCAC/SS cell. As shown in Figure 2(a), the metallic Li of 10 mA h cm ^-2^ is dissolved off, leading to a deep exposure of ant-nest-like network of the CaLi _2_ alloy as well as the carbon fiber primary framework. The microporous channels revealed in the high-magnification SEM image of Figure 2(d) are well interconnected. Certainly, this unique 3D architecture is helpful for liquid electrolyte penetration and efficient utilization of the active sites. Meanwhile, the cross-sectional SEM image in Figure 2(g) shows that the CaLi _2_ framework is uniformly nested in the CC as a secondary network among the carbon fibers. It is clear that this hybrid framework can facilitate ion diffusion and electron conduction. When 5 mA h cm ^-2^ Li was replated to LCAC (Figure 2(b)), Li nucleates and grows preferentially on the CaLi _2_ scaffold instead of on the carbon fibers, resulting in uniformly filling the interior space of the CaLi _2_ ant-nest-like channels without Li dendrites, which could be attributed to the lithiophilic nature and drastically reduced local current density of the CaLi _2_ scaffold. The cross-sectional SEM image depicts that the deposited Li metal is not aggravated on the electrode surface (Figure 2(h)), reflecting that the alloy framework can regulate Li deposition effectively. When the plating capacity reaches to 10 mA h cm ^-2^, the LCAC surface is uniformly enclosed with a dense Li layer (Figures 2(c) and 2(f)), and the void space in the bulk electrode is completely occupied (Figure 2(i)), indicating that the plating occurs firstly in the interior of the scaffold, and then gradually extends over the scaffold since the density of the deposited Li is slightly lower than that of the pristine Li metal. Herein, the Li nucleation and plating uniformity are significantly improved, which can prolongate the cycling lifetime greatly.

By deep contrast, LiC exhibits a quite different Li stripping/plating behavior. When 10 mA h cm ^-2^ Li is stripped from LiC (Fig. S8a), the Li metal touching with carbon fibers is initially etched off due to the high electrical conductivity of CC. As a result, a part of carbon fibers is exposed. Meanwhile, the Li dissolution produces a large number of holes with variable diameter, demonstrating the nonuniform behavior of Li stripping at the macroscopic scale. When plating 5 mA h cm ^-2^ Li back, the Li dendrites appear on the surface of the LiC electrode, covering the skeleton of CC completely (Fig. S6b). Apparently, it is very hard for the carbon fibers to induce Li deposition in a conformal way due to the poor affinity with Li. The growth of Li dendrites is exacerbated when fully plated back to 10 mA h cm ^-2^ Li, which can easily pierce separator and cause safety issue. Additionally, the voltage profiles of plating 1 mA h cm ^-2^ Li on the LiC or LCAC anode are demonstrated in Fig. S9. The LiC shows a large Li nucleation overpotential of 12.1 mV, and the LCAC displays a low overpotential of 8.8 mV during Li plating, indicating that LCAC host has much higher lithiophilicity than LiC.

**Figure 3:**
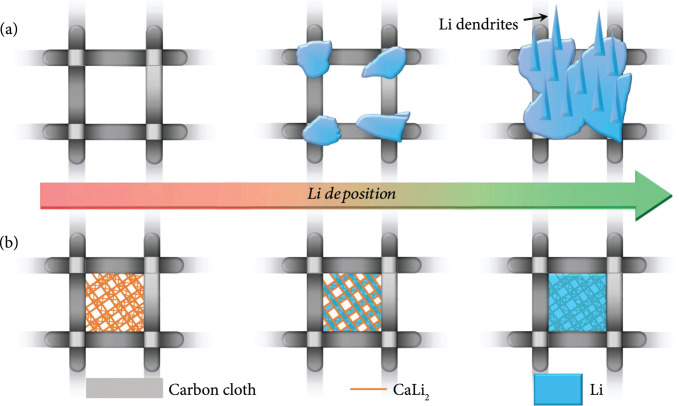
Schematic of the Li plating behavior on the (a) CC and (b) LCAC.

Figure 3 illustrates the difference in Li deposition behavior of the two electrodes schematically. The CC has limited specific surface area with poor wettability surface which leads to uneven Li nucleation and uncontrolled Li deposition, resulting the formation of Li dendrites. The Li metal deposited on CC shows uneven morphology with large size of Li dendrites after Li plating of 5 mA h cm ^-2^ (Figs. S10a-b). With increasing the areal capacity up to 10 mA h cm ^-2^, the plated Li is inevitably aggregated on the CC surface, leading to the greatly enhanced thickness of Li layer and the hardly visualized CC surface (Figure 3(a) and Fig S10c-d). As for the LCAC electrode, the ant-nest-like alloy network is decorated in the CC skeleton uniformly with irregular pores of approximately 2 ~ 3 *μ*m in size (Fig. S11), which effectively enlarges the specific surface area to reduce the local current density and homogenize the electric field distribution. Notably, the as-obtained ant-nest-like network is lithiophilic, which enables the reduced Li atoms preferentially to be condensed in the micropores of CaLi _2_ scaffold. Therefore, the plated Li metal fills up the interconnected pores of the alloy framework with flat appearance due to the uniform distribution of the CaLi _2_ subskeleton via the regulation of the Li ^+^ flux. The LCAC electrode is densely covered with a smooth Li layer and provides a dendrite-free morphology despite that the capacity of Li deposition is greatly promoted (Figure 3(b)).

**Figure 4:**
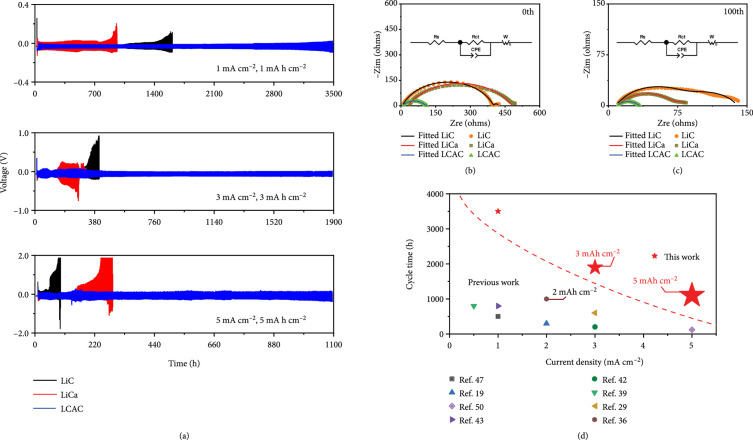
(a) The long-term cycling stabilities of symmetric cells with LCAC anode (blue), LiCa anode (red), or LiC anode (black) under various testing conditions (1 mA cm ^-2^, 1 mA h cm ^-2^; 3 mA cm ^-2^, 3 mA h cm ^-2^; 5 mA cm ^-2^, 5 mA h cm ^-2^). The impedance spectra, the fitted lines, and the equivalent circuit of LCAC, LiCa, and LiC symmetric cells (b) before and (c) after 100 cycles with 3 mA h cm ^-2^ at 3 mA cm ^-2^. (d) Cycling lifespans of symmetrical cells with LCAC anode compared to previously reported carbon-based Li composite anodes in terms of current density, cycle time, and areal capacity.

Galvanostatic charge/discharge measurements are conducted to evaluate the voltage hysteresis and long-term cycling stability of LCAC composite anode. The working electrode and counter electrode of each symmetric cell consist of two identical LCAC electrode sheets. To highlight the synergistic effect of the Li-Ca alloy scaffold and CC skeleton, LiC/LiC and LiCa/LiCa symmetric cells were assembled for comparison. The LiCa electrode on a stainless steel sheet was fabricated by adding Ca metal into the molten Li at 400°C at a molar ratio of 1 : 25 and then cooling down to room temperature. Figure 4(a) displays the voltage hysteresis-time curves of the symmetric cells at different current densities (1, 3, and 5 mA cm ^-2^) with the fixed areal capacity of 1, 3, or 5 mA h cm ^-2^, respectively. The overpotential during the Li stripping/plating process can reflect the electrochemical condition of Li metal anode. At the low current density of 1 mA cm ^-2^ with a low areal capacity of 1 mA h cm ^-2^, stable voltage profiles with small voltage hysteresis about 18 mV are observed in LCAC cell for more than 3500 h without any voltage increasing. In contrast, the cell with LiCa electrodes shows the worst performance, where a higher overpotential of 36 mV is recorded in initial cycles, and the polarization voltage increases sharply at 900 h, indicating that the accumulation of “dead Li” is serious, and the electrode structure is greatly damaged. Similarly, the cycling lifetime of the LiC-based symmetric cell is around 1500 h, which is much shorter than that of the LCAC anode. With the current density increases, the cells using LiC and LiCa anodes display larger voltage hysteresis and shorter lifespan (3 mA cm ^-2^: <400 h; 5 mA cm ^-2^: <150 h), which can be assigned to the formation of the fragile SEI layer and the rapid growth of Li dendrites under high current density. As a sharp contrast, the cell with LCAC composite anode exhibits longer cycling life of 2200 h and 800 h (Fig. S12), separately, which is also much higher than that of LiC and LiCa anodes. It is well known that the areal capacity of commercial Li-ion battery is higher than 3 mA h cm ^-2^, so the cycling areal capacity of the symmetrical cells is promoted to 3 mA h cm ^-2^. At a current density of 3 mA cm ^-2^, it is unexpectedly to illustrate that the LCAC anode achieves an ultralong lifespan of more than 1900 h with stable voltage hysteresis of ~50 mV. Unfortunately, the polarization of LiC anode and LiCa reaches 500 mV at 350 h and 250 h, respectively. Even when the current density and areal capacity are increased to a harsh condition (5 mA cm ^-2^, 5 mA h cm ^-2^), the LCAC anode still displays a smaller polarization and longer cycle life (~110 mV for 1100 h). By deep contrast, the LiC and LiCa symmetric cells exhibit a very large overpotential of ~1000 mV in a shorter time (LiC: 70 h; LCAC: 220 h), indicating severe Li dendrite growth, and the destroyed electrode structure occurs. As we know, Li metal anode has poor stability under carbonate-based electrolyte conditions. Figure S13 exhibits the long-term electrochemical performance of the Li composite electrodes with the electrolyte of 1 M lithium hexafluorophosphate (LiPF _6_) in ethylene carbonate (EC)/diethyl carbonate (DEC) (1 : 1 by volume) with 5% fluoroethylene carbonate (FEC). As shown in Fig. S13a-c, the LCAC anode has stable voltage profiles and longer cycling lifespan up to 2000, 650, and 260 h at the different current densities of 1, 3, and 5 mA cm ^-2^ with a low areal capacity of 1 mAh cm ^-2^, respectively, which is much higher than those of LiCa and LiC composite electrodes. While the areal capacity increases to 3 mAh cm ^-2^ (Fig. S13d), the cell using LCAC electrodes exhibits longer cycling lifetime for 400 h at 3 mA cm ^-2^, which can be ascribed to the hybrid structure of the LCAC composite for mitigating Li dendrites growth. In addition, electrochemical impedance spectroscopy (EIS) measurement is performed to reveal the interfacial stability in symmetric cell. As shown in Figure 4(b), the fitted charge transfer impedance ( $R_{\text{ct}}$ ) value of LCAC symmetric cell before cycling (98.3 *Ω*) was lower than that of LiCa (452.5 *Ω*) and LiC (393.8 *Ω*) symmetric cells. After 100 cycles under the condition of 3 mA h cm ^-2^ and 3 mA cm ^-2^, the fitted resistance of the LCAC cell decreases to 23.4 *Ω*, which is still lower than 58.7 *Ω* of the LiCa cell (58.7 *Ω*) and 64.9 *Ω* of the LiC cell, indicating that the LCAC symmetric cell can provide fast ion transfer kinetics in electrode/electrolyte interface with a more stable SEI layer. The superior electrochemical performance can be attributed to the lithiophilic CaLi _2_ secondary network and the flexible CC skeleton. The reduced surface tension of Li-Ca alloy results in good wettability toward CC, achieving the favorable synergistic effect. Additionally, compared with the published results for carbon-based Li composite anode (Figure 4(d) and Table S2), LCAC electrode can show the best cycling performance even at harsh conditions.

**Figure 5:**
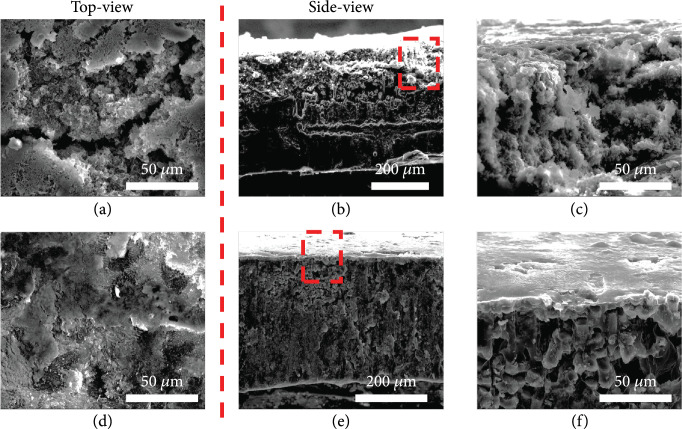
SEM images of the symmetric cells with the (a–c) LiC and (d–f) LCAC after 100 cycles at 3 mA cm ^-2^ with a fixed capacity of 3 mA h cm ^-2^.

The morphology evolution of the LCAC composite electrode and LiC electrode after repeated Li stripping/plating was investigated. Figures 5(a)– 5(c) exhibit the morphologies of the LiC electrode at 3 mA cm ^-2^ and 3 mA h cm ^-2^. In Figure 5(a), the surface of LiC electrode consists of plentiful Li dendrites, mossy Li, and cracked Li, which is ascribed to inhomogeneous Li deposition and uncontrolled Li growth. The cross-sectional SEM images (Figures 5(b) and 5(c)) reveal that the loose and crack Li deposition layer is as thick as of about 50 *μ*m with a rough surface. By deep contrast, the top-view SEM image of LCAC electrode (Figure 5(d)) reveals that LCAC provides smooth and dense Li deposition without any Li dendrites, indicating that the CaLi _2_ ant-nest-like framework of LCAC can form homogeneous Li ^+^ flux for dendrite-free morphology. The cross-sectional SEM image of the LCAC anode exhibits the steady structure and smooth surface (Figures 5(e) and 5(f)), further suggesting the effective regulation of Li stripping/plating by the favorable 3D skeleton host, which greatly improves the stability of the Li metal anode. In addition, LCAC electrode can significantly mitigate the volume change during cycling. As shown in Fig. S14a-b, the thickness of the LiC anode is dramatically increased from 304 *μ*m to 418 *μ*m after 100 cycles, in which the deposited Li layer is loose with a sharp boundary separating from the bulk Li domain (Fig. S14b). As a contrast, the thickness variation of the LCAC electrode before and after cycling is only ~5 *μ*m (Figs. S14c and d). Moreover, the reduced Li is plated in a compact way. Thus, the LCAC anode can alleviate volume fluctuation and guide Li deposition effectively during the long-term cycling process. Moreover, the XRD profile confirms that the main compositions of LCAC anode after 100 cycles are CaLi _2_ and Li, reflecting the favorable structure stability of CaLi _2_ framework nested in the CC matrix and ensuring long-term cycling lifespan (Fig. S15).

In order to gain a deeper insight on the effect of the CC as an external framework, the postmortem analysis of the dead LiCa and LCAC anodes was done by SEM imaging (Figure S16). As for the dead Li-Ca alloy electrode, a thick layer of Li dendrites with loose structure and microsized irregular particles appears, indicating that uneven Li deposition and unstable SEI formation occur during the repeated striping/plating. Apparently, the Li-Ca alloy electrode loses its functions even after a limited cycle for 250 h under a condition of 3 mA h cm ^-2^ and 3 mA cm ^-2^. On the contrary, the morphology of the dead LCAC electrode is completely different, showing a relatively flat and smooth surface which is composed of abundant nanosized particles instead of the dendritic granules with large size. It can be concluded that the synergistic effect between the external framework of carbon cloth and the internal skeleton of LiCa _x_ alloy network can effectively regulate Li nucleation and growth behavior, offering a stable interface with reduced polarization and expanded cyclic lifetime.

**Figure 6:**
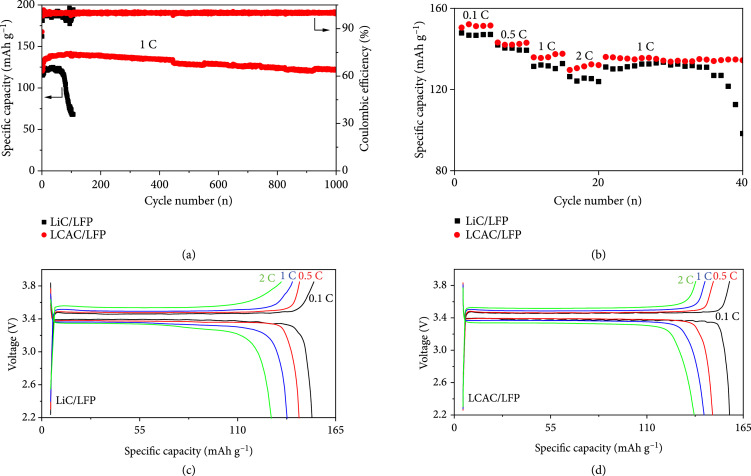
(a) The long-term cycling performance of the full cells of the LCAC/LFP or LiC/LFP performed at 1 C. (b) The specific capacity of two styles of the full cells at various rates from 0.1 to 2.0 C. The corresponding charge/discharge curves of full cells using (c) LiC and (d) LCAC as the anode at various rates in (b).

The LFP-based full cells were conducted for further eluidate the practical application possibility of LCAC composite anode. For comparison, LiC and LFP were paired as the full cell. Figure 6(a) shows the long-term cycling performance of LCAC/LFP and LiC/LFP full cells at a rate of 1 C. The slight increase in specific capacity of both cells at the initial stage can be attributed to the electrode activation process. The LCAC/LFP shows an initial charge capacity of 130 mA h g ^-1^, and the charge capacity of the LCAC/LFP still maintains 122 mA h g ^-1^ after 1000 cycles, exhibiting a capacity retaining ratio of 94% with stable CE. However, LiC/LFP delivers a lower initial specific capacity (117 mA h g ^-1^), which decays to 70 mA h g ^-1^ after only 100 cycles with sharp CE fluctuations. Furthermore, the N/P ratio of LiC and LCAC anodes in the full cell is lowered down to 2.0 and 1.6, respectively, which is really close to the setting value in the commercial Li-ion battery. As shown in Fig. S17, the LCAC/LFP cell exhibits a stable cycling up to 350 cycles with a capacity retention of ~92.8% at 1 C (2.45 mA cm ^-2^), which greatly outperforms the LiC/LFP cell (less than 30 cycles with a capacity retention of ~43.4%). It should be noted that the LCAC/LFP full cell with low N/P ratio displays a slightly shorter cycling lifespan, implying that the Li striping/plating behavior in the well-designed LCAC hosting architecture is not perfectly done. The rate performances of LCAC/LFP and LiC/LFP are demonstrated in Figure 6(d). The LCAC/LFP exhibits a higher specific capacity of cells 152, 142, 138, and 132 mA h g ^-1^ at 0.1, 0.5, 1, and 2 C, respectively, which are higher than that of the LiC/LFP cells (147, 139, 132, and 124 mA h g ^-1^). These results demonstrate that the production of dead Li and Li dendrites severely reduces the Li utilization and charge transfer process in LiC/LFP cell. In contrast, Li metal can be uniformly deposited into the well-designed alloy microporous network, and a stable SEI layer is apt to be formed, ensuring excellent charge transfer process and high Li utilization of LCAC/LFP full cells.

## 3. Conclusion

In summary, we have developed a composite anode material with the lithiophilic CaLi _2_ ant-nest-like network by a simple thermal infusion strategy to enable the uniform Li deposition into the interior space. Owing to the improved wettability of Li-Ca alloy against CC, Li-Ca alloy can easily spread into the CC substrate for building the secondary network. The interconnected channels of the CaLi _2_ ant-nest-like framework contribute to further reduce the local current density, confine the Li deposition, and enhance the affinity with Li, which can induce uniform Li nucleation and deposition. Moreover, the mechanical stability of the CaLi _2_ with MgZn _2_ type Laves phase structure is significantly enhanced by the utilization of CC, eliminating the volume change of the electrode and facilitating the formation of a robust SEI during the repeated cycles. As a result, LCAC can exhibit a dendrite-free Li deposition behavior and delivers long-term lifespan for 1100 h under the harsh condition of 5 mA cm ^-2^ and 5 mA h cm ^-2^. Remarkably, benefiting from the favorable cycling reversibility and fast charge transfer kinetics, the excellent full cell performance assembled with high areal capacity cathodes has been achieved. We believe that LCAC has great potential of application for the next generation of high-energy-density Li metal batteries.

## 4. Materials and Methods

### 4.1. Preparation of LCAC Electrode

The LCAC electrode was fabricated by infusing molten Li-Ca alloy into a commercial carbon cloth (CC). The CC was cut into pellets with a diameter of 14 mm. Then, the thermal infusion method was performed in an argon-filled glove box (Mikrouna, ${\text{O}}_{2}\leq 0.01$ ppm, ${\text{H}}_{2}\text{O}\leq 0.01$ ppm). First of all, the mixture of Ca metal (Aladdin, 99.5%) and Li foil (Chengdu Denway Newtype Metal Material Co., Ltd., 99.5%) was weighed with an atomic ratio of 1 : 25 and subsequently heated up to 400°C on a stainless steel (SS) sheet. The Ca metal was uniformly dissolved into molten Li and finally obtained liquid Li-Ca alloy during a period of time. Then, the CC pellet was put on the liquid alloy, which was rapidly infused into the CC. Following that, the LCAC composite electrode was prepared by cooling to the room temperature. The LiC composite electrode was produced by infiltrating molten Li into CC disks at the same conditions except that the temperature is 500°C.

### 4.2. Material Characterization

The phase composition of LCAC was performed by X-ray diffraction (XRD, Cu K *α* radiation) at a step size of 5° min ^-1^ with the 2 *θ* range from 10 to 90°. The morphology and structure of the anode were characterized on a field emission scanning electron microscopy (FE-SEM, Hitachi, S3400N) at 20 kV.

### 4.3. Electrochemical Evaluation

To test the electrochemical performance of anodes, the galvanostatic cycling performances were utilized by the instrument CT2001A LAND system at constant temperature of 25°C. On a CHI 660C analyzer, EIS measurements were carried out over the frequency from 100 mHz to 100 kHz with an amplitude of 5 mV. For the symmetrical cells, two identical LCAC electrodes were used as both anode and cathode and assembled in CR2032 coin cell. The electrolyte with 1 M lithium bistrifluoro-methanesulfonyl imide (LiTFSI) in 1, 2-dimethoxyethane (DME) and 1, 3-dioxolane (DOL) mixed at 1 : 1 with 2 wt% LiNO _3_ as an additive was employed unless otherwise specified. The amount of the electrolyte in each cell was fixed at 100 *μ*L. For the full cells, LiFePO4 (LFP) powder as the active material, super P and polyvinylidene fluoride (PVDF, binder) were blended at a mass ratio of 8 : 1 : 1 and dissolved in N-methyl-2-pyrrolidone (NMP) to form uniform slurry. The mixed slurry was coated on a carbon-coated aluminum (Al) foil and further dried at 110°C in a vacuum oven to evaporate the excess solvent and moisture for 12 h. Then, the obtained composite was used as the cathode, and the average areal loading of LFP is 17.2 mg cm ^-2^ (2.45 mAh cm ^-2^) with a diameter of 12 mm. The Celgard 2325 was performed as the separator sheet with a diameter of 19 mm, and the thickness of the LCAC anode is about 330 *μ*m with a diameter of 14 mm. The as-assembled LFP-based full cells were cycled between 2.2 and 3.85 V. When fabricating Li composite with low Li content, a tiny amount of molten metal was transferred on stainless steel substrate, where both of the liquid metal and the substrate were kept at 400°C. Then, the CC sheet was contacted with the molten metal, which was immediately moved back and forth so as to make the adsorbed liquid metal distributed uniformly in the CC sheet. Here, the infiltrated Li content is roughly controlled by the contacting time. In the full cell test, the low N/P ratios of LiC-based and LCAC-based full cells were about 2.0 and 1.6 with a short touching time of ~3 s, separately.

## Data Availability

All data needed to evaluate the conclusions in the paper are present in the paper and/or the Supplementary Materials. Additional data related to this paper may be requested from the authors.

## Supplementary Materials

Supplementary Materials Figure S1: time-lapse images of (a) liquid Li and (b) liquid Li-Ca alloy infusion process on the CC at 400°C. Figure S2: wettability of molten Li-Ca and Li-Ag alloys with different molar ratios on CC substrates. Figure S3: XRD pattern of the LCAC. Figure S4: voltage profile of stripping Li from LCAC electrode to -2 V versus Li ^+^/Li. Figure S5: the (a, b) top-view and (c, d) side-view SEM images of the as-prepared LiC electrode, where the insert images are the corresponding optical photographs. Figure S6: (a) The SEM images of LCAC electrode after stripping 20 mA h cm ^-2^ Li. (b) The corresponding high-magnification SEM image of the marked area. Figure S7: (a) The SEM images of LCAC electrode after Li fully stripping. (b) The corresponding high-magnification SEM image of the marked area. (c) The XRD pattern of delithiated LCAC. Figure S8: The SEM images of LiC electrode after (a) stripping 10 mA h cm ^-2^ Li, (b) plating 5 mA h cm ^-2^ Li back, and (c) plating 10 mA h cm ^-2^ Li back. Figure S9: the voltage profile during plating 1 mA h cm ^-2^ Li on the (a) LiC and (b) LCAC anode. Figure S10: SEM images after Li plating of (a, b) 5 mA h cm ^-2^ and (c, d) 10 mA h cm ^-2^ on CC. Figure S11: SEM image of ant-nest-like structure of LCAC after removing free Li. Figure S12: electrochemical characterization of symmetric cells at charge/discharge of (a) 1 mA h cm ^-2^ and 3 mA cm ^-2^ and (b) 1 mA h cm ^-2^ and 5 mA cm ^-2^. Figure S13: The long-term electrochemical performance of the symmetric cells in the carbonated electrolytes under the condition of (a) 1 mA cm ^-2^, 1 mA h cm ^-2^; (b) 3 mA cm ^-2^, 3 mA h cm ^-2^; (c) 5 mA cm ^-2^, 1 mA h cm ^-2^; (d) 3 mA cm ^-2^, 3 mA h cm ^-2^. Figure S14: the cross-sectional SEM images of (a, b) LiC and (c, d) LCAC anode (a, c) before and (b, d) after 100 cycles at 3 mA cm ^-2^ with a fixed capacity of 3 mA h cm ^-2^. Figure S15: XRD pattern of LCAC anode after 100 cycles at a condition of 3 mA cm ^-2^ and 3 mA h cm ^-2^. Figure S16: SEM images of (a, b) LiCa anode and (c, d) LCAC anode after long-term cycling up to failure. Figure S17: cycling performance of the full cells with two styles of anodes at 1 C with low N/P ratio. Table S1: data used to calculate theoretical specific capacity of LCAC electrode. Table S2: comparison of the LCAC anode in this work with other carbon-based scaffolds reported in recent publications.
