# Supplementary material for: Li-Ca Alloy Composite Anode with Ant-Nest-Like Lithiophilic Channels in Carbon Cloth Enabling High-Performance Li Metal Batteries
Source: Research (Wash D C). 2022 Jun 23;2022:9843093. doi: 10.34133/2022/9843093 (PMC11412416; doi:10.34133/2022/9843093)
Supplement: Supplementary Materials — Figure S1: time-lapse images of (a) liquid Li and (b) liquid Li-Ca alloy infusion process on the CC at 400°C. Figure S2: wettability of molten Li-Ca and Li-Ag alloys with different molar ratios on CC substrates. Figure S3: XRD pattern of the LCAC. Figure S4: voltage profile of stripping Li from LCAC electrode to -2 V versus Li +/Li. Figure S5: the (a, b) top-view and (c, d) side-view SEM images of the as-prepared LiC electrode, where the insert images are the corresponding optical photographs. Figure S6: (a) The SEM images of LCAC electrode after stripping 20 mA h cm -2 Li. (b) The corresponding high-magnification SEM image of the marked area. Figure S7: (a) The SEM images of LCAC electrode after Li fully stripping. (b) The corresponding high-magnification SEM image of the marked area. (c) The XRD pattern of delithiated LCAC. Figure S8: The SEM images of LiC electrode after (a) stripping 10 mA h cm -2 Li, (b) plating 5 mA h cm -2 Li back, and (c) plating 10 mA h cm -2 Li back. Figure S9: the voltage profile during plating 1 mA h cm -2 Li on the (a) LiC and (b) LCAC anode. Figure S10: SEM images after Li plating of (a, b) 5 mA h cm -2 and (c, d) 10 mA h cm -2 on CC. Figure S11: SEM image of ant-nest-like structure of LCAC after removing free Li. Figure S12: electrochemical characterization of symmetric cells at charge/discharge of (a) 1 mA h cm -2 and 3 mA cm -2 and (b) 1 mA h cm -2 and 5 mA cm -2. Figure S13: The long-term electrochemical performance of the symmetric cells in the carbonated electrolytes under the condition of (a) 1 mA cm -2, 1 mA h cm -2; (b) 3 mA cm -2, 3 mA h cm -2; (c) 5 mA cm -2, 1 mA h cm -2; (d) 3 mA cm -2, 3 mA h cm -2. Figure S14: the cross-sectional SEM images of (a, b) LiC and (c, d) LCAC anode (a, c) before and (b, d) after 100 cycles at 3 mA cm -2 with a fixed capacity of 3 mA h cm -2. Figure S15: XRD pattern of LCAC anode after 100 cycles at a condition of 3 mA cm -2 and 3 mA h cm -2. Figure S16: SEM images of (a, b) LiCa anode and (c, [file 9843093.f1.docx]

**Li-Ca Alloy Composite Anode with Ant-nest-like Lithiophilic Channels in Carbon Cloth Enabling High-performance Li Metal Batteries**

**Authors**

Zihao Wang^1,2^, Yuchi Liu^1,2^, Jianxiong Xing^1,2^, Zhicui Song^1,2^, Aijun Zhou^1,2^, Wei Zou^3^, Fu Zhou^3^, Jingze Li^1,2^*

**Affiliations**

^1^ School of Materials and Energy, University of Electronic Science and Technology of China, Chengdu 611731, P. R. China

^2^ Yangtze Delta Region Institute (Huzhou), University of Electronic Science and Technology of China, Huzhou 313001, P. R. China

^3^ Research and Development Center, Tianqi Lithium Co., Ltd., Chengdu 610093, P. R. China

Correspondence should be addressed to Jingze Li; lijingze@uestc.edu.cn


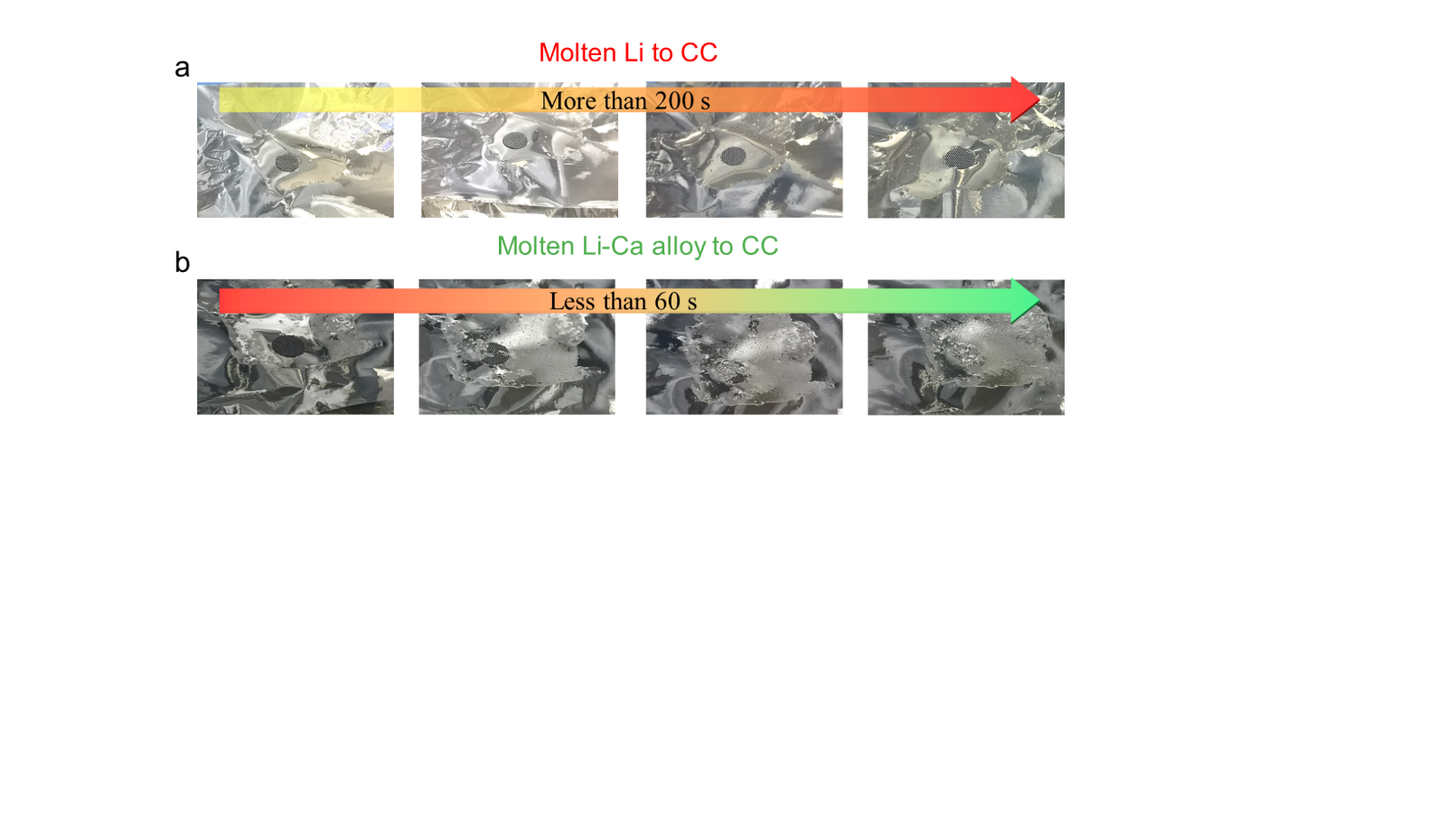


Figure S1. Time-lapse images of (a) liquid Li and (b) liquid Li-Ca alloy infusion process on the CC at 400 ºC.


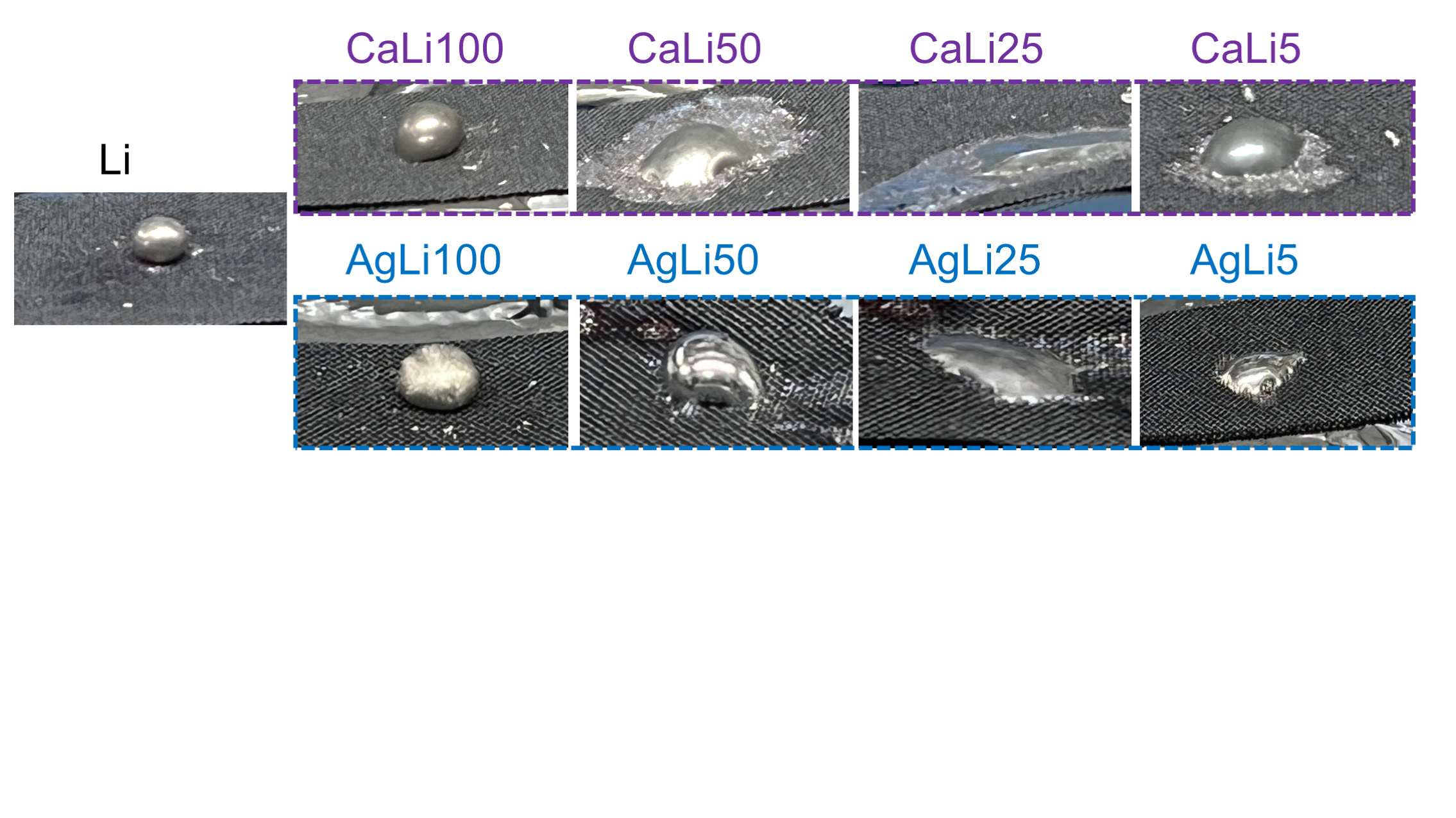


Figure S2. Wettability of molten Li-Ca and Li-Ag alloys with different molar ratios on CC substrates.



Figure S3. XRD pattern of the LCAC.



Figure S4. Voltage profile of stripping Li from LCAC electrode to -2 V versus Li^+^/Li.


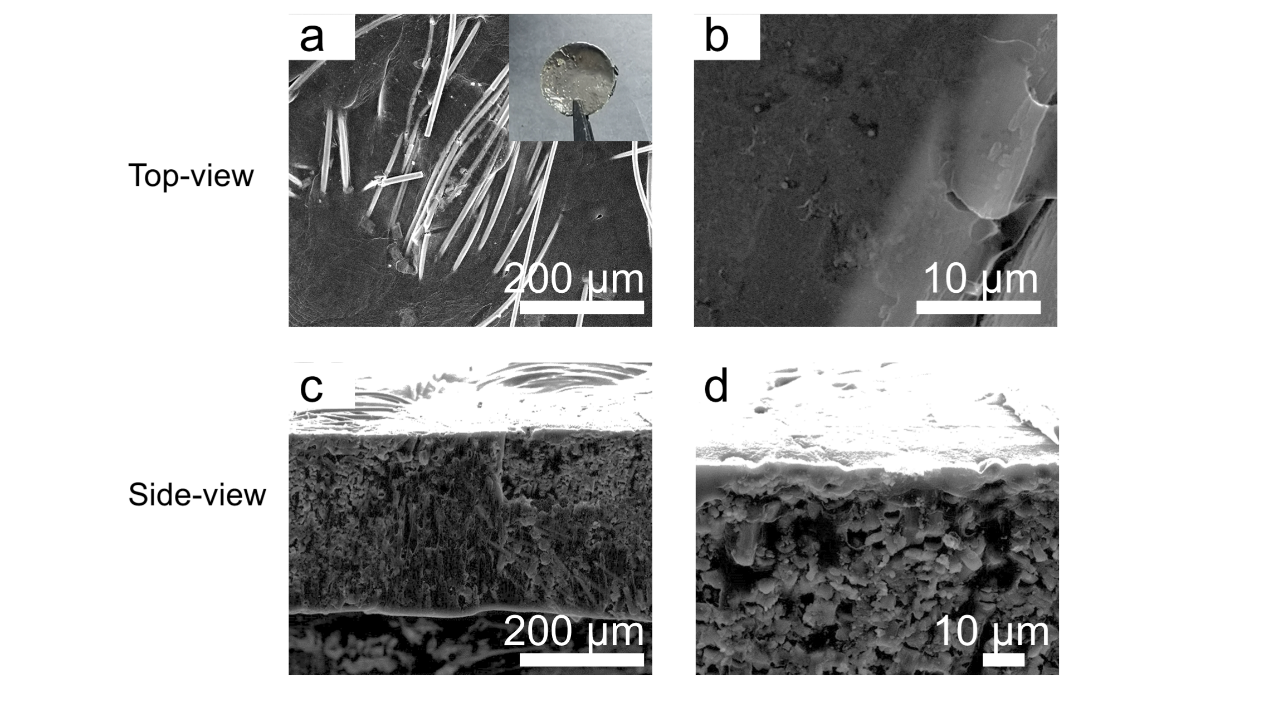


Figure S5. The (a, b) top-view and (c, d) side-view SEM images of the as-prepared LiC electrode, where the insert images are the corresponding optical photographs.


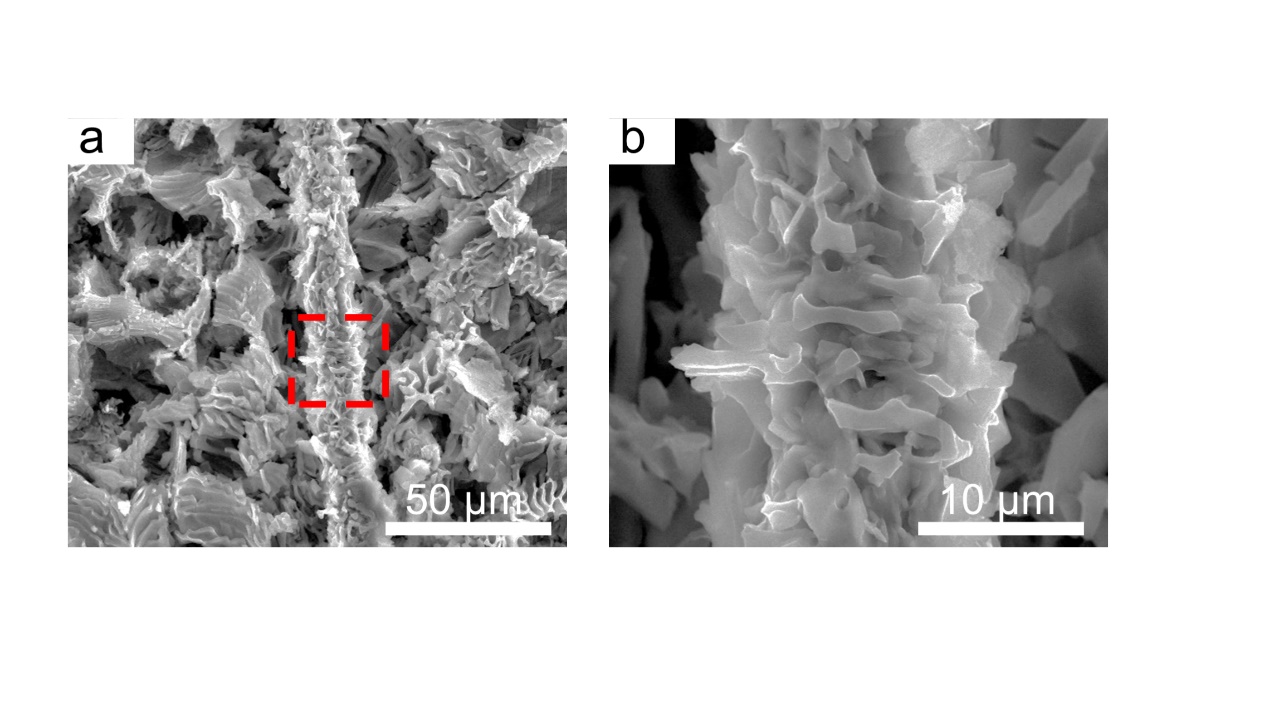


Figure S6. (a) The SEM images of LCAC electrode after stripping 20 mA h cm^-2^ Li. (b) The corresponding high-magnification SEM image of the marked area.


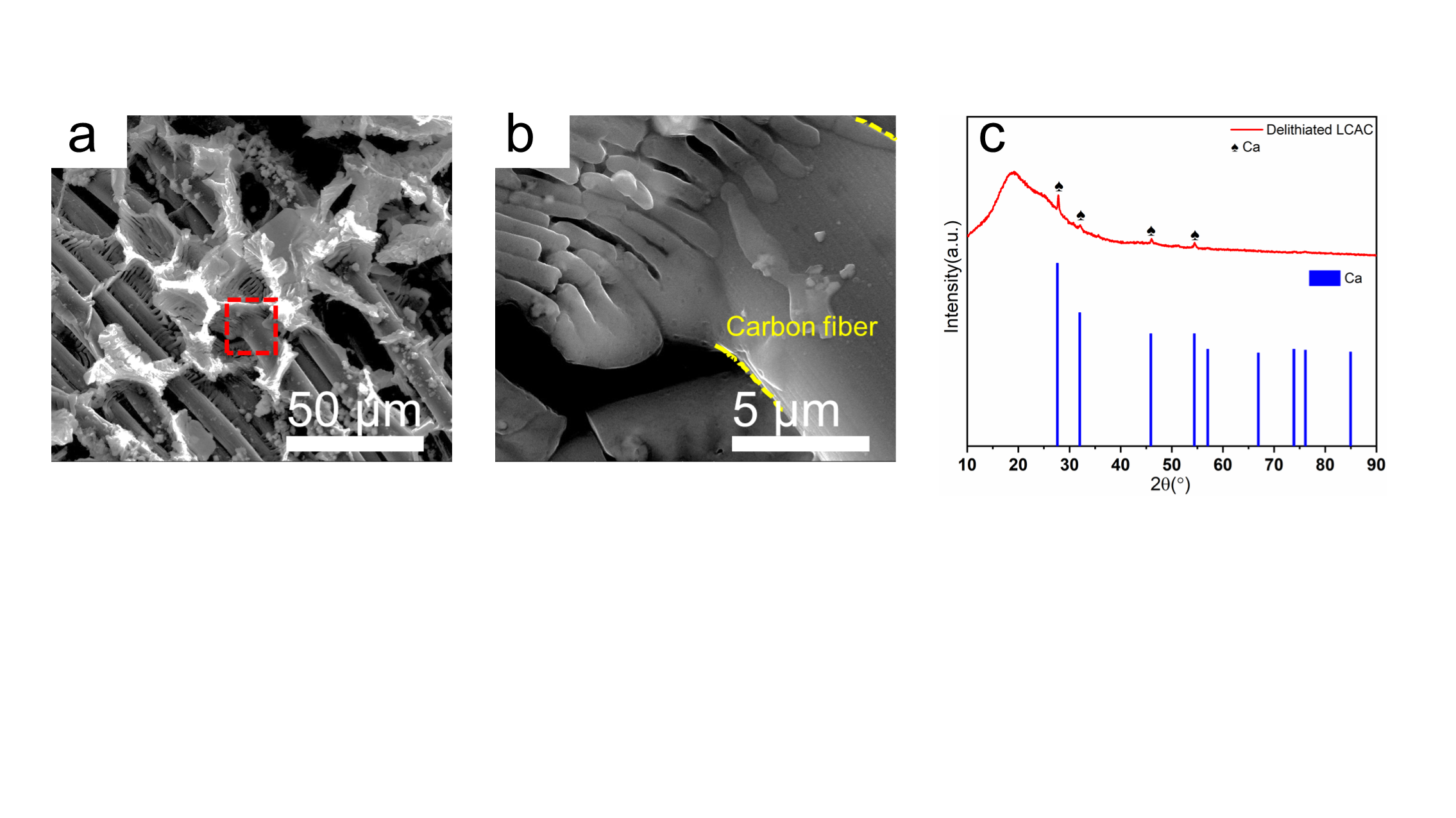


Figure S7. (a) The SEM images of LCAC electrode after Li fully stripping. (b) The corresponding high-magnification SEM image of the marked area. (c) the XRD pattern of delithiated LCAC.


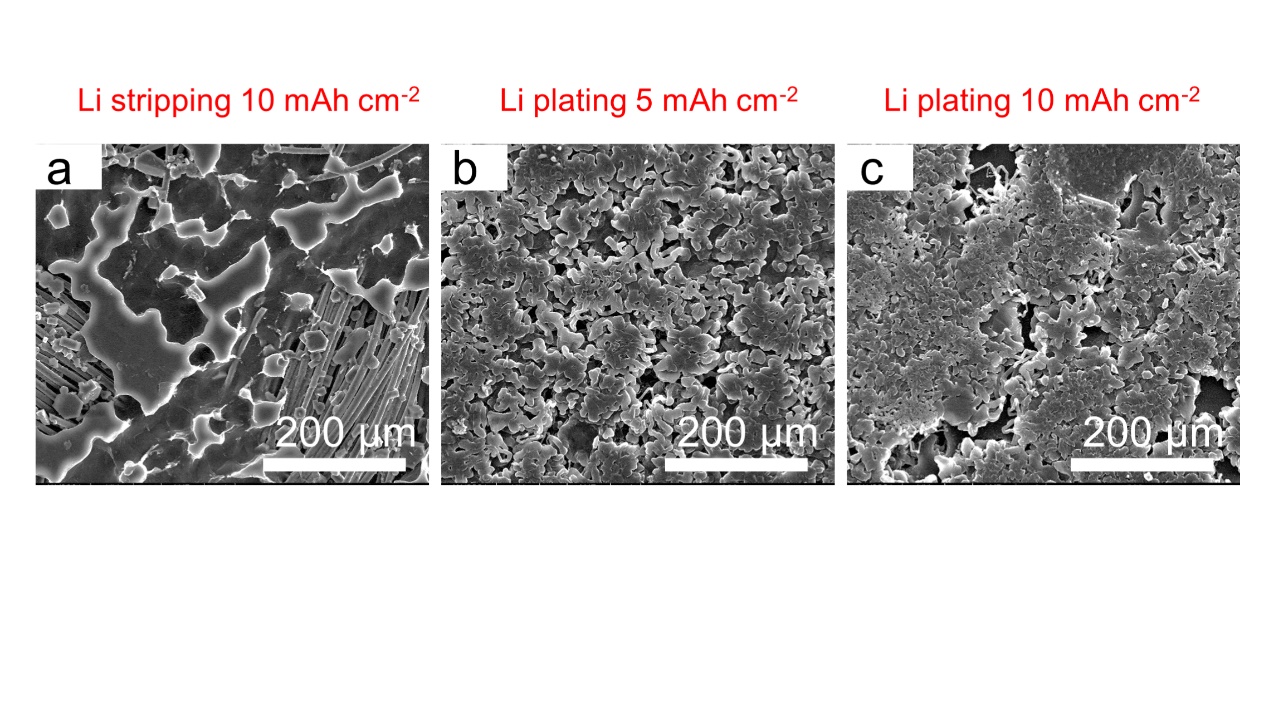


Figure S8. The SEM images of LiC electrode after (a) stripping 10 mA h cm^-2^ Li, (b) plating 5 mA h cm^-2^ Li back, and (c) plating 10 mA h cm^-2^ Li back.


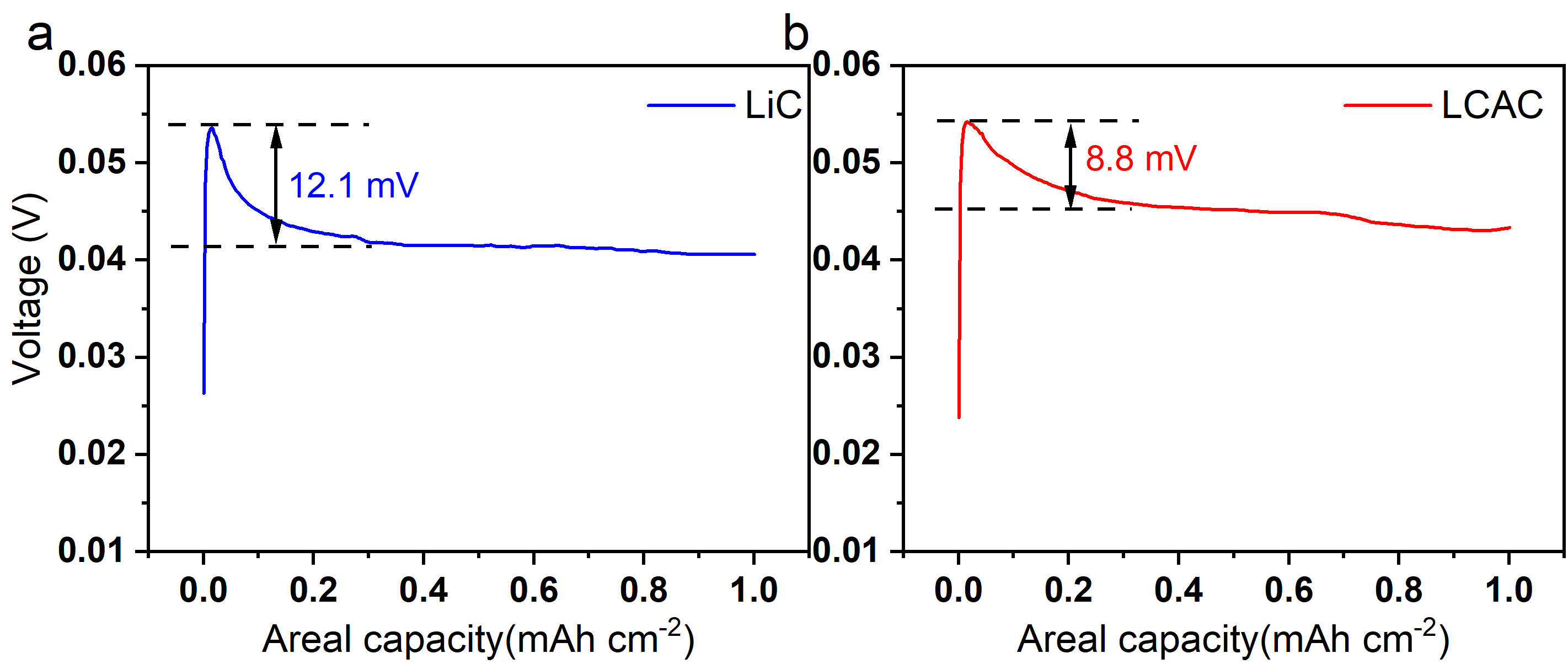


Figure S9. The voltage profile during plating 1 mA h cm^‑2^ Li on the (a) LiC and (b) LCAC anode.


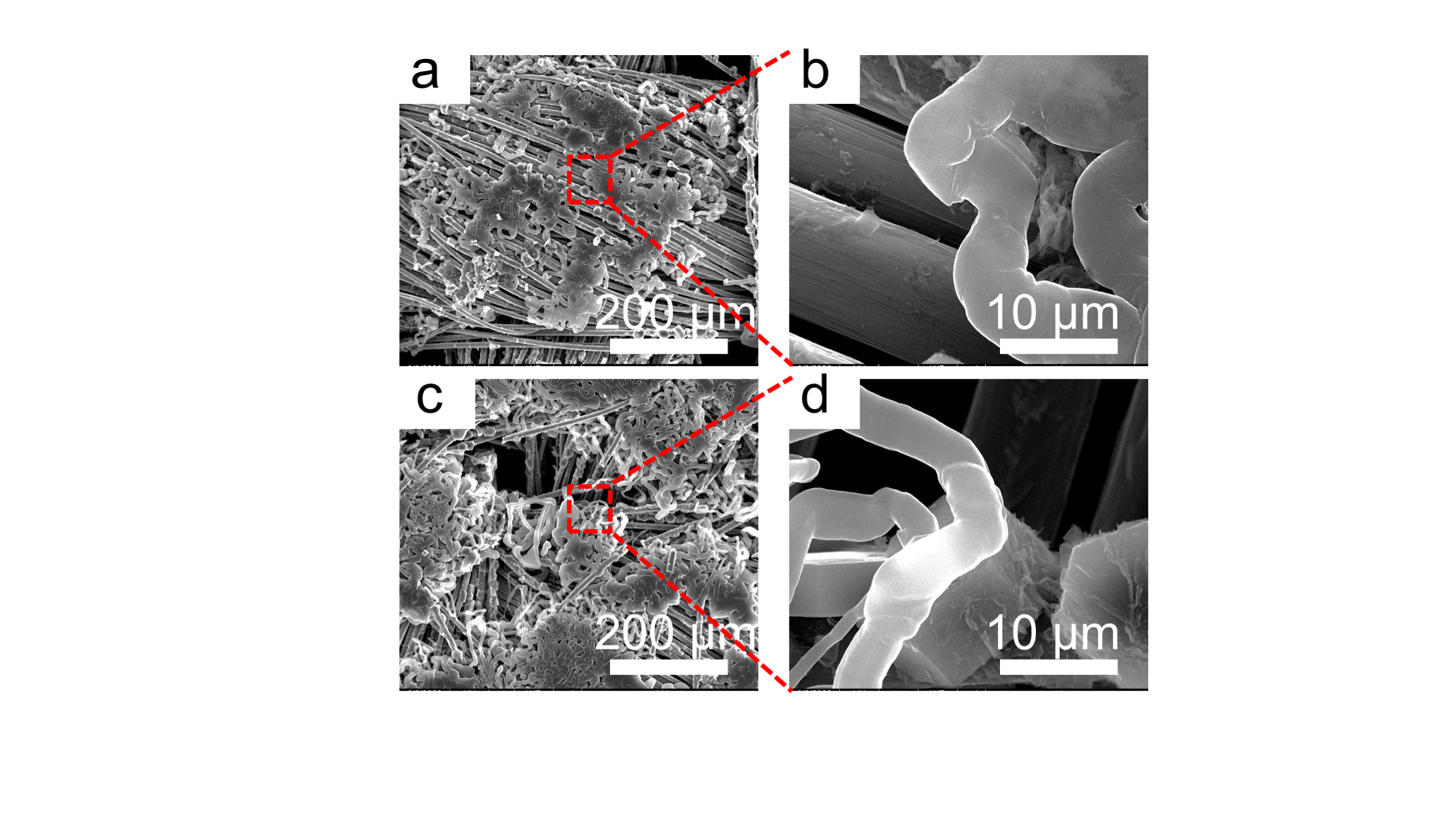


Figure S10. SEM images after Li plating of (a, b) 5 mA h cm^-2^ and (c, d) 10 mA h cm^-2^ on CC.


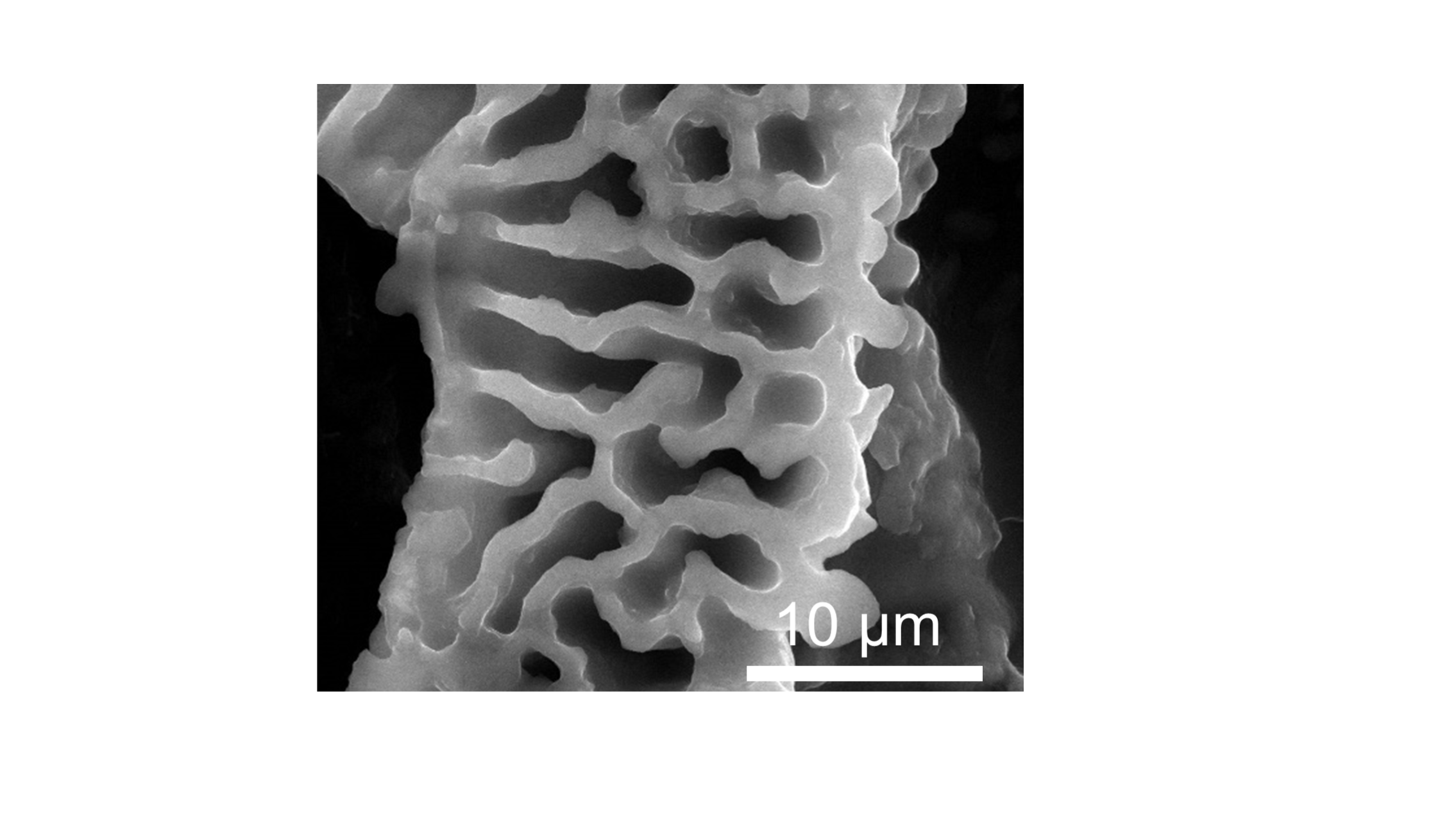


Figure S11. SEM image of ant-nest-like structure of LCAC after removing free Li.





Figure S12. Electrochemical characterization of symmetric cells at charge/discharge of (a) 1 mA h cm^-2^ and 3 mA cm^-2^, (b) 1 mA h cm^-2^ and 5 mA cm^-2^.



 Figure S13. The long-term electrochemical performance of the symmetric cells in the carbonated electrolytes under the condition of (a) 1 mA cm^-2^, 1 mA h cm^-2^; (b) 3 mA cm^-2^, 3 mA h cm^-2^; (c) 5 mA cm^-2^, 1 mA h cm^-2^; (d) 3 mA cm^-2^, 3 mA h cm^-2^.


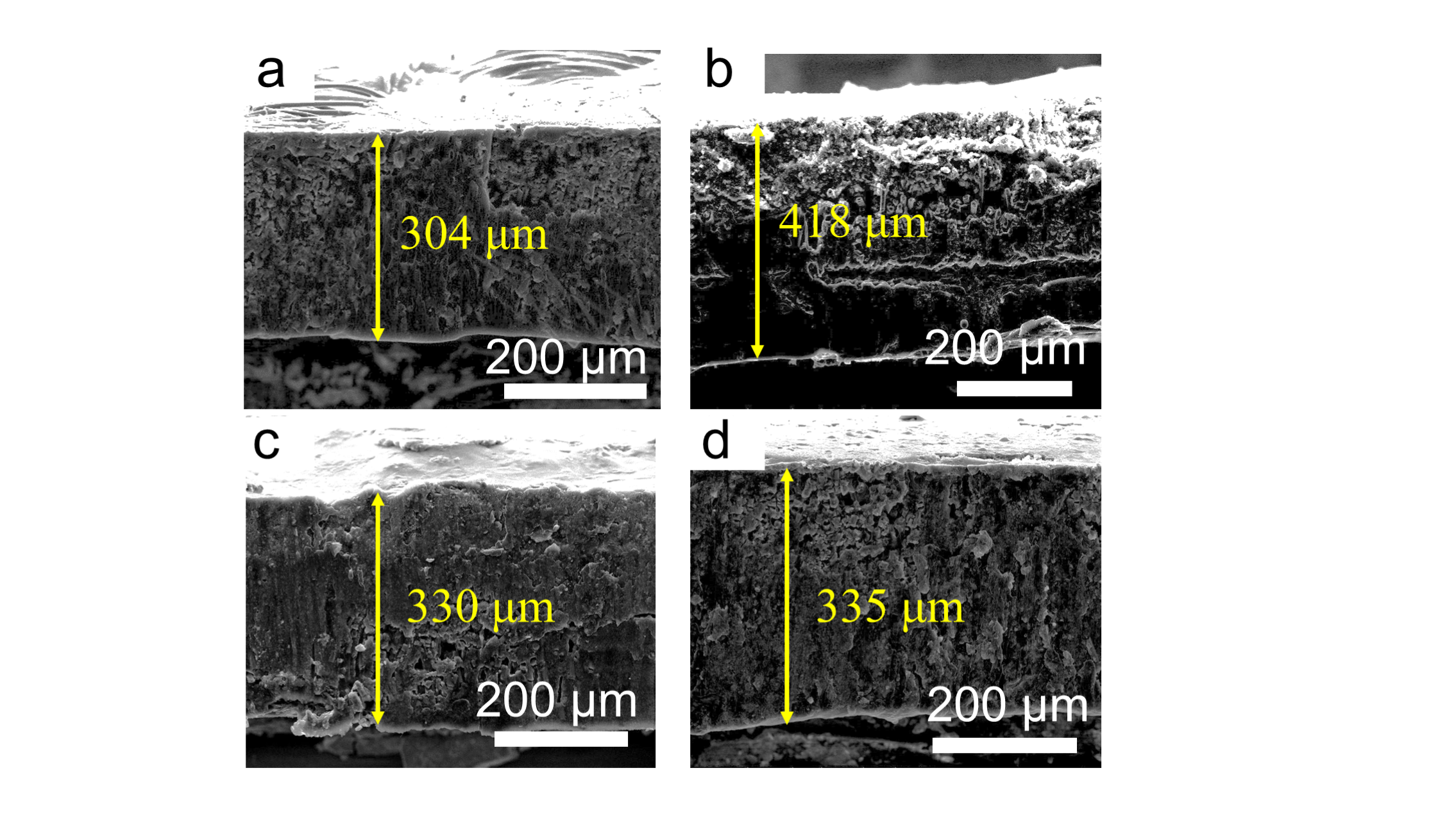


Figure S14. The cross-sectional SEM images of (a, b) LiC and (c, d) LCAC anode (a, c) before and (b, d) after 100 cycles at 3 mA cm^-2^ with a fixed capacity of 3 mA h cm^-2^.





Figure S15. XRD pattern of LCAC anode after 100 cycles at a condition of 3 mA cm^-2^, 3 mA h cm^-2^.


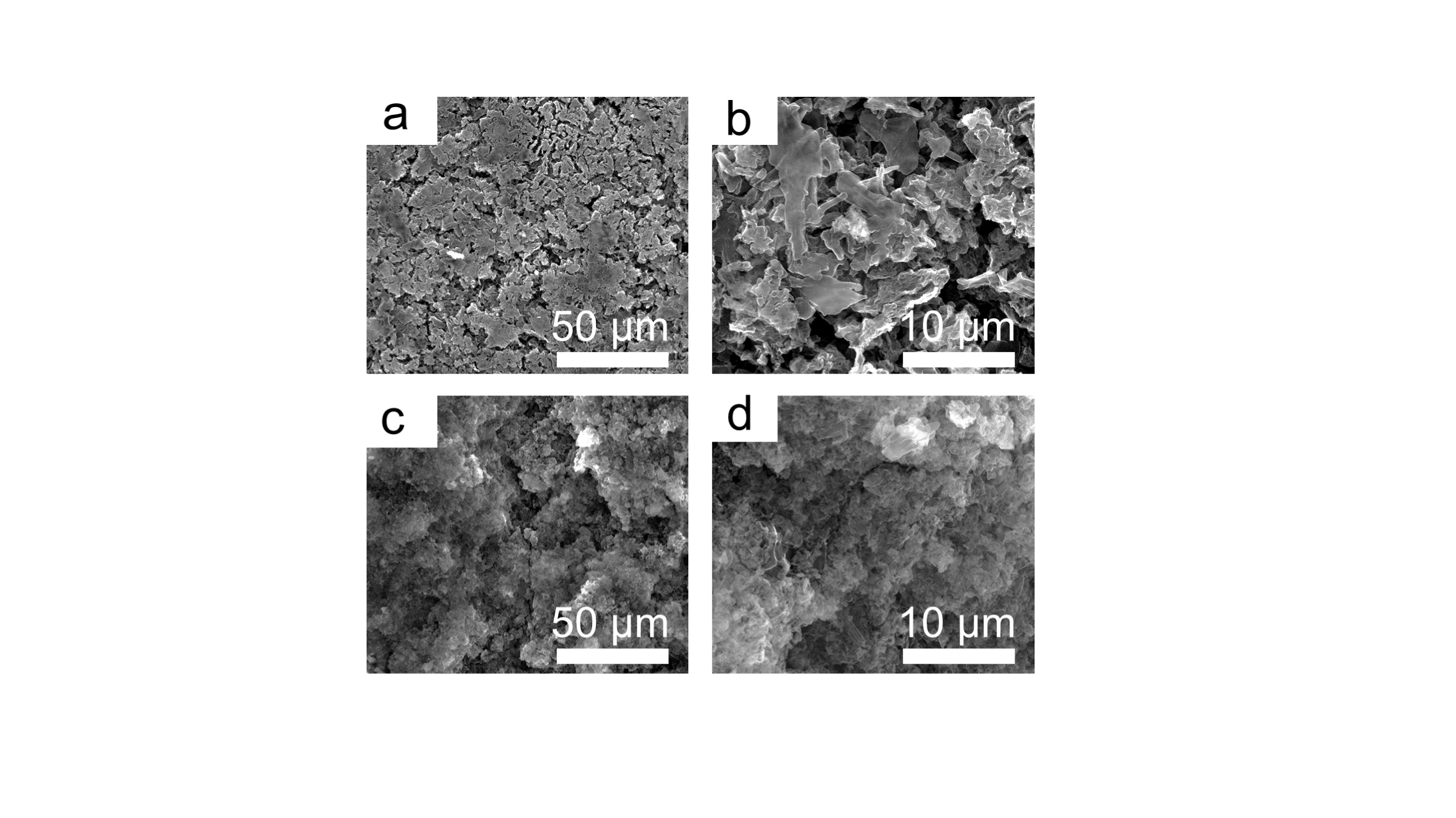


Figure S16 SEM images of (a, b) LiCa anode and (c, d) LCAC anode after long-term cycling up to failure.


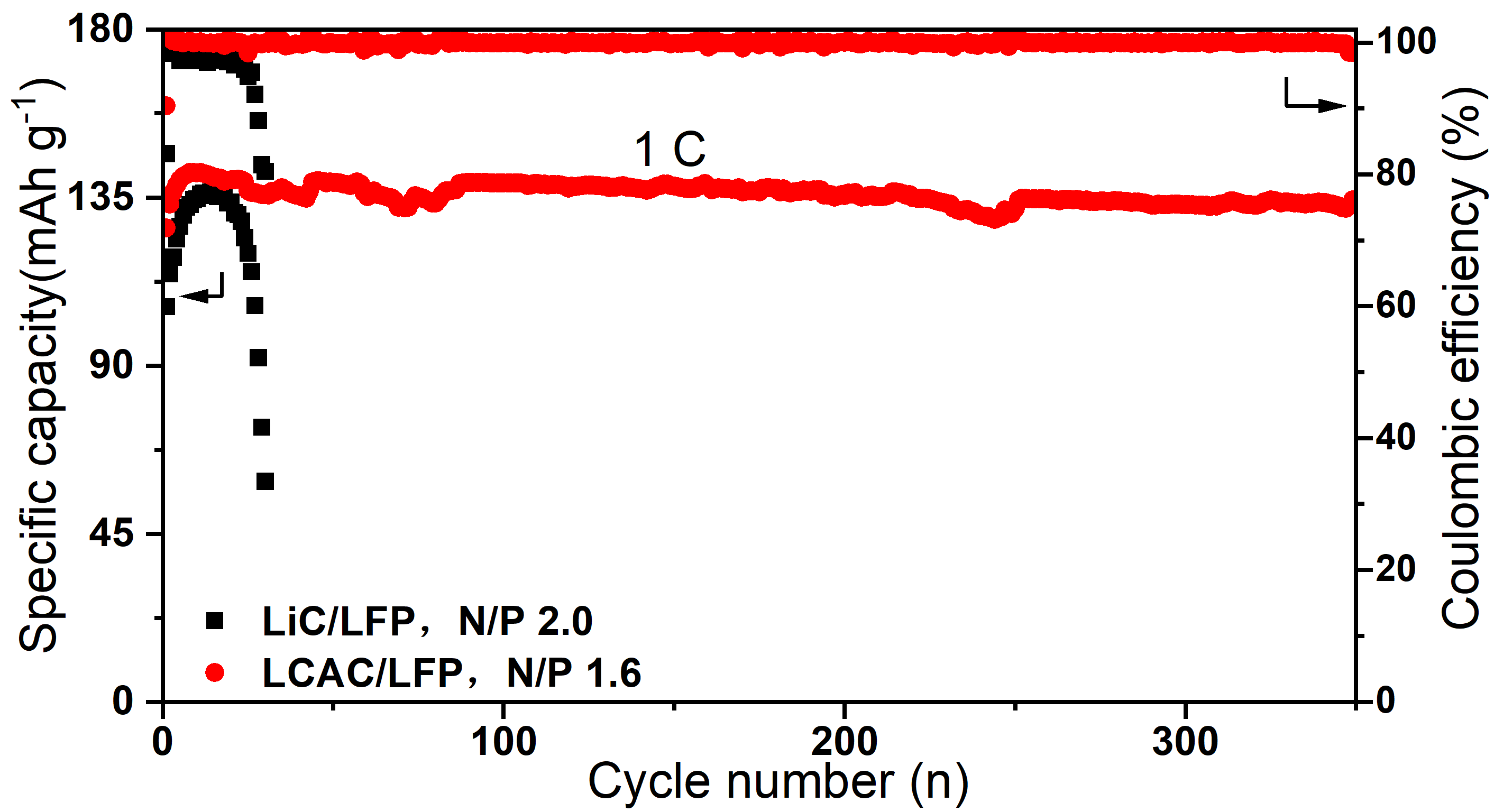


Figure. S17 Cycling performance of the full cells with two styles of anodes at 1 C with low N/P ratio.

Table S1. Data used to calculate theoretical specific capacity of LCAC electrode.

| Substance | Average quality (mg) | wt (%) |
| --- | --- | --- |
| LCAC | 26.5 | 100 |
| CC | 9.5 | 35.85 |
| Ca (CaLi_2_) | 3.19 (4.29) | 12.04 (16.19) |
| Li (metallic Li) | 13.81 (12.71) | 52.11 (47.96) |

LCAC theoretical specific capacity = m(Li)/m(LCAC) ×3860 = 13.81 ÷ 26.5 × 3860 ≈ 2011 mA h g^-1^

Table S2. Comparison of the LCAC anode in this work with other carbon-based scaffolds reported in recent publications.

|  | Electrolyte Components | Current density  (mA cm^-2^) | Capacity of Li  (mAh cm^-2^) | Time |
| --- | --- | --- | --- | --- |
| CNTs^[1]^ | 1M LiTFSI in DOL/DME with 1% LiNO_3_ | 1 | 1 | 2000 h |
| CC/CNT^[2]^ | 1M LiTFSI in DOL/DME with 1% LiNO_3_ | 1  2  5 | 1  1  1 | 500 h  500 h  500 h |
| TiC/C/Li^[3]^ | 1M LiTFSI in DOL/DME with 1% LiNO_3_ | 3 | 1 | 200 h |
| GCF^[4]^ | 1M LiTFSI in DOL/DME with 1% LiNO_3_ | 1  2 | 1  1 | 1000 h  300 h |
| MGCN^[5]^ | 1M LiTFSI in DOL/DME with 2% LiNO_3_ | 0.5 | 1 | 800 h |
| Li-CC@ZnO^[6]^ | 1M LiTFSI in DOL/DME | 5 | 1 | 120 h |
| 3D GF^[7]^ | 1M LiTFSI in DOL/DME with 1% LiNO_3_ | 1 | 1 | 1000 h |
| LiCF^[8]^ | 1M LiTFSI in DOL/DME with 5% LiNO_3_ | 1  5 | 1  5 | 1000 h  80 h |
| NGCF@Li^[9]^ | 1M LiTFSI in DOL/DME with 2% LiNO_3_ | 2  3 | 1  1 | 1200 h  600 h |
| 3DCP^[10]^ | 1M LiTFSI in DOL/DME with 1% LiNO_3_ | 2 | 2 | 1000 h |
| Co-CS/Li^[11]^ | 1M LiTFSI in DOL/DME | 1 | 1 | 800 h |
| CNF-Sn^[12]^ | 1M LiTFSI in DOL/DME with 0.2M LiNO_3_ | 0.5  1 | 1  1 | 850 h  650 h |
| Li@HPTCF^[13]^ | 1M LiTFSI in DOL/DME with 2% LiNO_3_ | 1 | 1 | 300 h |
| **This work** | **1M LiTFSI in DOL/DME with 2% LiNO_3_** | **1**  **3**  **3**  **5** | **1**  **1**  **3**  **5** | **3500 h**  **2200 h**  **1900 h**  **1100 h** |

Reference

[1] L. Ye; M. Liao; H. Sun; Y. Yang; C. Tang; Y. Zhao; L. Wang; Y. Xu; L. Zhang; B. Wang; F. Xu; X. Sun; Y. Zhang; H. Dai; P. G. Bruce; H. Peng. Angew Chem Int Ed Engl. 58(2019) 2437-2442.<http://doi.org/10.1002/anie.201814324>

[2] F. Liu; R. Xu; Z. Hu; S. Ye; S. Zeng; Y. Yao; S. Li; Y. Yu. Small. 15(2019) e1803734.<http://doi.org/10.1002/smll.201803734>

[3] S. Liu; X. Xia; Y. Zhong; S. Deng; Z. Yao; L. Zhang; X.-B. Cheng; X. Wang; Q. Zhang; J. Tu. Advanced Energy Materials. 8(2018).<http://doi.org/10.1002/aenm.201702322>

[4] T. T. Zuo; X. W. Wu; C. P. Yang; Y. X. Yin; H. Ye; N. W. Li; Y. G. Guo. Adv Mater. 29(2017).<http://doi.org/10.1002/adma.201700389>

[5] Z. Li; X. Li; L. Zhou; Z. Xiao; S. Zhou; X. Zhang; L. Li; L. Zhi. Nano Energy. 49(2018) 179-185.<http://doi.org/10.1016/j.nanoen.2018.04.040>

[6] X. Wang; Z. Pan; Y. Wu; X. Ding; X. Hong; G. Xu; M. Liu; Y. Zhang; W. Li. Nano Research. 12(2018) 525-529.<http://doi.org/10.1007/s12274-018-2245-z>

[7] L. Pan; Z. Luo; Y. Zhang; W. Chen; Z. Zhao; Y. Li; J. Wan; D. Yu; H. He; D. Wang. ACS Appl Mater Interfaces. 11(2019) 44383-44389.<http://doi.org/10.1021/acsami.9b17108>

[8] P. Shi; T. Li; R. Zhang; X. Shen; X. B. Cheng; R. Xu; J. Q. Huang; X. R. Chen; H. Liu; Q. Zhang. Adv Mater. 31(2019) e1807131.<http://doi.org/10.1002/adma.201807131>

[9] L. Liu; Y. X. Yin; J. Y. Li; S. H. Wang; Y. G. Guo; L. J. Wan. Adv Mater. 30(2018).<http://doi.org/10.1002/adma.201706216>

[10] X. Zhou; W. Huang; C. Shi; K. Wang; R. Zhang; J. Guo; Y. Wen; S. Zhang; Q. Wang; L. Huang; J. Li; X. Zhou; S. Sun. ACS Appl Mater Interfaces. 10(2018) 35296-35305.<http://doi.org/10.1021/acsami.8b13506>

[11] S. Li; Q. Liu; J. Zhou; T. Pan; L. Gao; W. Zhang; L. Fan; Y. Lu. Advanced Functional Materials. 29(2019).<http://doi.org/10.1002/adfm.201808847>

[12] T. Liu; J. Hu; C. Li; Y. Wang. ACS Applied Energy Materials. 2(2019) 4379-4388.<http://doi.org/10.1021/acsaem.9b00573>

[13] W. Cai; G. Li; D. Luo; G. Xiao; S. Zhu; Y. Zhao; Z. Chen; Y. Zhu; Y. Qian. Advanced Energy Materials. 8(2018).<http://doi.org/10.1002/aenm.201802561>
